# Exploring the relationship between climate-related exposures and oral health outcomes in Pakistan: A community-based cross-sectional study

**DOI:** 10.1371/journal.pgph.0006190

**Published:** 2026-03-27

**Authors:** Kawish Syed, Syed Midhat Batool, Rabia Khatoon, Hafiz Muhammad Owais Nasim, Abid Rahim, Kaisar Khan, Farhad Ali Khattak, Muhammad Ilyas, Sofia Shehzad

**Affiliations:** 1 Consultant Periodontist, Dent Ortho, Peshawar, Pakistan; 2 Bakhtawar Ameen Medical and Dental College, Multan, Pakistan; 3 Sardar Begum Dental College, Gandhara University, Peshawar, Pakistan; 4 CMH Lahore Medical College and Institute of Dentistry, Lahore, Pakistan; 5 Khyber Medical Institute of Dental Sciences, Khyber Medical University, Peshawar, Pakistan; 6 Khyber Medical University, Peshawar, Pakistan; 7 District Headquarters Hospital, Charsadda, Pakistan; Kisii University, KENYA

## Abstract

Climate change is increasingly recognized as a contextual determinant of health in low- and middle-income countries (LMICs). Climate-related stressors such as water scarcity and food insecurity may influence oral health indirectly by disrupting hygiene practices, dietary patterns, and access to care. However, epidemiological evidence examining these relationships remains limited, particularly in resource-limited settings. This study aimed to examine the association between selected climate-related exposures and oral health outcomes among adults residing in climate-vulnerable districts of Pakistan. A cross-sectional analytical study was conducted among 422 adults residing in Charsadda and Peshawar districts of Khyber Pakhtunkhwa, Pakistan. Climate-related exposures assessed over the preceding 12 months included water scarcity, food insecurity, heat stress, and climate-related displacement. Oral health outcomes were evaluated using standardized clinical measures: the Decayed, Missing, and Filled Teeth (DMFT) index and the Community Periodontal Index (CPI). Multivariable binary logistic regression models were used to examine associations between climate-related exposures and oral health outcomes after adjusting for socio-demographic and behavioral confounders. Principal component analysis was performed to explore clustering of environmental and behavioral risk factors. Among the 422 adults, 61.4% had high caries (DMFT ≥ 3) and 47.6% had periodontal disease (CPI ≥ 3). Water scarcity (49.8%) and food insecurity (45.5%) were frequently reported. In adjusted analysis, water scarcity (AOR 1.89;95% CI 1.23-2.9) and food insecurity (AOR 1.74; 95% CI 1.12-2.69) were independently associated with high caries. For periodontal disease, water scarcity (AOR 1.67; 95% CI 1.12-2.48) and food insecurity (AOR 1.59;95% CI 1.05-2.41) were independently associated. Principal component analysis identified clustering of environmental stressors with hygiene- and nutrition-related vulnerabilities. Water scarcity and food insecurity were associated with adverse oral health outcomes in climate-vulnerable districts of Khyber Pakhtunkhwa. Integrating climate resilience with preventive oral health strategies may help in the reduction of emerging environmental health inequalities.

## Introduction

Climate change is increasingly recognized as a major global public health challenge, with effects that extend beyond environmental degradation to shape the social, economic, and health conditions in which populations live. Low- and middle-income countries (LMICs) are disproportionately affected by climate-related hazards due to limited adaptive capacity, high dependence on climate-sensitive livelihoods, and pre-existing structural inequalities [1,2]. Rising temperatures, recurrent flooding, water scarcity, food insecurity, and climate-related displacement have become defining features of daily life for millions of people in vulnerable regions, exacerbating health inequities and straining already fragile health systems [3–5].

While the health impacts of climate change have been extensively documented in relation to infectious diseases, undernutrition, maternal and child health, and heat-related morbidity and mortality, oral health has received comparatively limited attention within climate and environmental health research [6]. Oral diseases, particularly dental caries and periodontal disease, are among the most prevalent non-communicable diseases globally and contribute substantially to disability-adjusted life years [7]. Despite being largely preventable, these conditions disproportionately affect socioeconomically disadvantaged populations, reflecting inequities in access to resources, healthcare, and healthy living environments [8].

Oral health is strongly influenced by social, behavioral, and environmental determinants, including access to safe water, adequate nutrition, sanitation, education, and healthcare services [7,9]. Climate-related stressors can disrupt these determinants through interconnected pathways. Water scarcity may limit routine oral hygiene practices, food insecurity may increase reliance on inexpensive cariogenic diets, and displacement may interrupt access to dental care services [10–12]. Chronic stress associated with climate vulnerability may further influence health behaviors and care-seeking patterns [13]. In this context, climate change should be conceptualized not as a direct biological cause of oral disease, but as a structural driver shaping exposure to established oral health risk factors.

The concept of climate-related exposure has therefore emerged as a pragmatic framework for examining health impacts of climate change at the population level [14]. Unlike climate change itself, which operates over long temporal and spatial scales, climate-related exposures capture proximal stressors such as water insecurity, heat stress, displacement, and food shortages that are measurable in epidemiological studies [15]. This distinction is particularly important in cross-sectional research designs, where long-term climatic trends cannot be directly assessed. Focusing on climate-related exposures allows for the examination of associations without implying causality or temporal change.

Despite growing recognition of the importance of integrating oral health into public health and equity agendas, empirical evidence linking climate-related exposures to oral health outcomes remains scarce [16]. Existing literature is dominated by conceptual frameworks, narrative reviews, and policy discussions, with relatively few population-based studies assessing measurable oral health outcomes in climate-vulnerable settings [17]. Recent scoping reviews have highlighted the lack of epidemiological studies examining associations between climate-related stressors and standard oral health indices such as the Decayed, Missing, and Filled Teeth (DMFT) index and the Community Periodontal Index (CPI), particularly in LMIC contexts [18].

Pakistan provides a highly relevant context in which to explore these associations. The country is consistently ranked among the most climate-vulnerable nations globally, facing recurrent floods, prolonged heatwaves, water scarcity, and food insecurity [19,20]. These challenges intersect with rapid urbanization, socioeconomic inequality, and limited health system capacity. Provinces such as Khyber Pakhtunkhwa have experienced repeated environmental shocks that disproportionately affect low-income and peri-urban communities, many of which already face barriers to accessing basic healthcare services, including oral health care [21].

Understanding the relationship between climate-related exposures and oral health requires careful consideration of confounding factors. Socioeconomic status, education, income, occupation, and place of residence are strongly associated with both exposure to environmental stressors and oral health outcomes [22]. Climate-related exposures rarely occur in isolation; water scarcity, food insecurity, displacement, and poor hygiene practices often co-exist and interact synergistically [23]. Identifying such clustering patterns can enhance understanding of vulnerability profiles and inform integrated public health interventions.

Oral health indicators in Pakistan reveal a substantial unmet burden of disease. High prevalence of untreated dental caries and periodontal disease has been reported across age groups, particularly among socioeconomically disadvantaged populations [24,25]. Access to preventive and restorative dental services remains limited, and oral health is often excluded from primary healthcare and public health planning [26]. In climate-vulnerable settings, these challenges may be further exacerbated by disruptions to water supply, food systems, and healthcare access. Yet, empirical data linking these environmental stressors to oral health outcomes remain limited.

This study was conducted to examine the association between climate-related stressors and oral health outcomes among adults residing in selected districts of Khyber Pakhtunkhwa, Pakistan. Specifically, we assessed whether household exposure to water scarcity, food insecurity and climate related displacement were associated with high caries burden and moderate to severe periodontal disease. We further evaluated whether these associations persisted after adjustment for socio-demographic and behavioural factors. By addressing these questions within the community-based sample, this study contributes empirical evidence to the limited epidemiological literature on climate-sensitive oral health in resource-limited settings.

## Methods

### Study design

This study employed a cross-sectional analytical design to examine associations between climate-related exposures and oral health outcomes among adults residing in selected districts of Khyber Pakhtunkhwa, Pakistan. A cross-sectional approach was selected as an appropriate epidemiological design for generating population-level evidence in a resource-limited setting where longitudinal climate–health data are not routinely available. The study was not designed to assess long-term climate change trajectories or establish causal relationships; rather, it focused on individual-level exposure to climate-related stressors experienced within a defined time frame and their association with contemporaneous oral health status.

### Study setting

The study was conducted in the districts of Charsadda and Peshawar, located in Khyber Pakhtunkhwa province, Pakistan. These districts were selected to reflect variation in urbanization, population density, and exposure to environmental stressors. Peshawar represents a predominantly urban setting with comparatively better access to healthcare infrastructure, but represents a densely populated urban area facing rising temperatures, air pollution and increasing urban stress while Charsadda reflects a peri-urban and rural context characterized by higher vulnerability to flooding, water insecurity, and limited public services. Khyber Pakhtunkhwa is widely recognized as a climate-vulnerable region due to recurrent floods, rising temperatures, seasonal water shortages, and climate-related displacement.

### Study population

The study population consisted of adults aged 18 years and above residing in the selected districts at the time of data collection. Adults were selected because oral health outcomes such as dental caries and periodontal disease reflect cumulative exposure to behavioral, social, and environmental determinants over time.

### Inclusion and exclusion criteria

Individuals were eligible for inclusion if they were aged 18 years or older, had resided continuously in the study area for at least 12 months prior to data collection, and were willing to provide written informed consent. Individuals with severe systemic illness affecting oral health (head & neck cancer, radiotherapy and chemotherapy patients, Sjogren Syndrome), those undergoing active orthodontic treatment or extensive dental rehabilitation, and pregnant women with medical complications requiring specialized care were excluded to reduce clinical heterogeneity and avoid confounding of oral disease outcomes.

### Justification for residency criterion

A minimum residency period of 12 months was required to ensure exposure to seasonal and recurrent climate-related stressors, including fluctuations in water availability, heat exposure, and food access. This criterion aligns with public health frameworks that conceptualize climate-related exposure as proximal environmental stressors rather than long-term climatic change. Although oral diseases often develop over extended periods, disruptions in hygiene practices, dietary patterns, and access to care within one year can meaningfully influence oral health status, particularly periodontal conditions.

### Sample size calculation

The sample size was calculated using the standard formula for estimating proportions in cross-sectional studies. Assuming 50% prevalence of poor oral health, a 95% confidence interval and 5% margin of error, the minimum sample size was 384. After adjusting for a 10% non-response rate, the final sample size was 422, equally divided between Charsadda (n = 211) and Peshawar (n = 211).

### Sampling procedure

The study used a multistage community-based sampling framework within selected districts. In each district, urban and rural union councils were selected. While district selection was purposive to capture climate-vulnerable contexts, household-level participant selection was systematic and standardized. Within each household, a single participant was selected using the Kish method (Kish L. Survey Sampling. New York: Wiley; 1965). Stratification ensured proportional representation of age, gender and socioeconomic status across both districts.

### Data collecting instrument

Data were collected using a structured interviewer-administered questionnaire adapted from WHO Oral Health Survey Methods (5th edition) [27], and published climate vulnerability and food insecurity indicators. The questionnaire included sections on demographic (age, gender, income, education and occupation), climate exposure (flooding, drought, water quality, displacement), oral hygiene practices (frequency of brushing, use of toothpaste, access to clean water), dietary habit and self-reported oral symptoms (pain, bleeding, dryness, swelling). The questionnaire was translated into Pashto and Urdu. Clinical oral examination was conducted in accordance with the World Health Organization Oral Health Survey Guidelines [27]. Dentists calibrated for inter-examiner reliability (Kappa >0.85) assessed DMFT, CPI and presence of oral lesions. Standard infection control measures were observed. Examinations were conducted in designated community health centers and mobile dental units where necessary.

### Questionnaire validation

The questionnaire was pilot-tested among 30 adults from a non-study area with similar socio-demographic characteristics. Internal consistency of the exposure and behavioral domains demonstrated acceptable reliability, with a Cronbach’s alpha of 0.81. Minor modifications were made to improve clarity and cultural appropriateness.

### Measurement of climate-related exposure

Environmental exposure was quantified through the Climate Exposure Index, created by scoring self-reported exposure to: 1) extreme heat events, 2) water scarcity, 3) displacement due to flooding, 4) crop failure/food shortage and 5) poor air quality. Each item was scored on a 0–3 scale based on frequency, with a composite index ranging from 0 to 15. Participants were categorized into low (0–5), moderate (6–10) or high (11–15) climate exposure risk.

### Measurement of oral health behaviors

Oral hygiene behaviors were assessed using self-reported measures, including frequency of tooth brushing, use of toothpaste, use of additional hygiene aids, dietary sugar consumption, and dental service utilization. Poor oral hygiene practice was defined as brushing less than twice daily or inconsistent use of toothpaste.

### Clinical oral examination

Clinical oral examinations were conducted by trained dental professionals following WHO criteria. Dental caries experience was assessed using the Decayed, Missing, and Filled Teeth (DMFT) index, while periodontal status was evaluated using the Community Periodontal Index (CPI). Calibration exercises were performed prior to data collection to ensure consistency.

### Variables/operational definitions

#### Resource-limited settings and Low- and Middle-Income Countries (LMICs).

Resource-limited settings are defined as local contexts characterized by constrained healthcare infrastructure, limited access to dental services, and socioeconomic disadvantage, whereas LMICs refer to a broader global economic classification.

***Caries burden:*** High caries burden is defined as (DMFT ≥3) consistent with population-based adult oral health studies and periodontal disease (CPI ≥ 3).

***Climate-vulnerable communities:*** Climate-vulnerable communities are defined as populations experiencing increased exposure and limited adaptive capacity to climate-related stressors, such as water scarcity, food insecurity, heat stress, and displacement, within the context of socioeconomic disadvantage.

#### Climate-sensitive areas.

The term climate-sensitive refers to geographic regions where livelihoods, infrastructure, and health outcomes are particularly sensitive to climate variability and extreme weather events.

#### Climate change, environmental change, and climate-related exposure.

Climate change refers to long-term changes in climatic patterns; environmental change is used only in a general contextual sense of the environment; and climate-related exposures refer specifically to measurable individual- or household-level experiences assessed in this study.

*Confounding variables* included age, sex, education, income, residence, and oral hygiene practices.

### Statistical analysis

Data were entered and analyzed using SPSS software version 23.0. Descriptive statistics summarized participant characteristics. Continuous variables were summarized using means and standard deviations and categorical variables were summarized using frequencies and percentages. Two binary outcome variables were defined: high caries (DMFT ≥3) burden and moderate to severe periodontal disease(CPI ≥ 3).

Bivariate logistic regression analysis was used to estimate crude odds ratios (ORs) and 95% confidence intervals (CIs) for the association between the independent variable and the outcomes of high caries (DMFT ≥3) and periodontal disease (CPI ≥ 3).

Multivariable logistic regression models were constructed separately for each outcome to estimate adjusted odds ratios (AORs) and 95% CIs. All socio-demographic variables (age, sex, education, income, residence and district) and behavioral factors (brushing frequency and fluoride toothpaste use) were included in the model. Climate-related stressors (water scarcity, food insecurity and displacement) were entered simultaneously to assess their independent associations. Multicollinearity was assessed using variance inflation factors (VIF) with VIF > 10 considered indicative of significant collinearity and model fit was evaluated using the Hosmer-Lemeshow goodness of fit test. Statistical significance was defined as a two sided p-value <0.5 Missing data were assessed using Little’s MCAR test. Variables with less than 5% missing were addressed using list-wise deletion. Principal component analysis was performed to explore clustering of climate-related and hygiene-related variables.

### Ethical considerations

Ethical clearance was obtained from the Ethical Review Committee of the Gandhara University, Peshawar (No.GU/Ethical Committee/2025/314). Written informed consent was obtained from all participants. Community engagement included stakeholder briefing with local health authorities, dissemination of oral health pamphlets and referral of patients with urgent oral conditions to local clinics.

### Quality control

Quality control involved weekly monitoring visits, random re-checks of 10% of clinical exams for reliability and daily backup of electronic data.

## Results

A total of 422 participants were included in the study. The mean age of the study population was 36.2 ± 11.5 years, ranging from 18 to 72 years. Females constituted 52.1% (n = 220) of the sample, while males accounted for 47.86% (n = 202). Over 60% of respondents reported a monthly household income below PKR 25,000 (≈ USD 90), indicating that the study population predominantly comprised low-income households. Educational attainment varied, with 34.6% having no formal education, 29.1% completing primary education, 21.3% secondary education, and only 15.0% attaining post-secondary education. Unemployment or informal labor was reported by 48.2% of participants.

### Oral health status

The overall mean DMFT score was 3.95 ± 2.6. High caries burden (DMFT ≥3) was observed in 61.4% (n = 259) of participants. Periodontal assessment showed that 47.6% (n = 201) had CPI scores ≥3, indicative of periodontal disease. Both DMFT and CPI scores were significantly higher among individuals with lower income, lower education, and poor oral hygiene practices. More than 60% of all participants reported brushing only once per day or less. Poor oral hygiene practices were more prevalent in Charsadda (62%) than in Peshawar (54%). The fluoride toothpaste was low (35% overall) with a significant urban-rural gap.

### Climate-related exposures

Water scarcity was reported by 49.8% (n = 210) of participants, food insecurity by 45.5% (n = 192), heat stress by 52.1% (n = 220), and climate-related displacement by 18.2% (n = 77). Overall, 68.7% (n = 290) of participants experienced at least one climate-related stressor in the preceding 12 months. Co-occurrence of multiple stressors was common, particularly among low-income households in Charsadda.

### Bivariate analysis

In bivariate logistic regression analysis, several socio-demographic, behavioral, and climate-related exposures were associated with oral health outcomes as shown in Table 1.

**Table 1:** Bivariate logistic regression analysis (Crude OR).

Variable	Category	High Caries OR (95% CI)	p-value	CPI3 OR (95% CI)	p-value
District	Charsadda vs Peshawar	1.15 (0.83–1.59)	0.392	1.20 (0.87–1.65)	0.268
Residence	Rural vs Urban	1.90 (1.27–2.85)	0.002	1.54 (1.10–2.17)	0.012
Age Group	≥45 vs 18–29	1.58 (1.01–2.48)	0.046	1.92 (1.33–2.77)	<0.001
Sex	Female vs Male	1.08 (0.75–1.56)	0.673	1.12 (0.79–1.59)	0.523
Education	<Secondary vs ≥Secondary	2.15 (1.43–3.22)	<0.001	1.62 (1.13–2.32)	0.008
Income	<25,000 PKR vs ≥ 25,000	1.68 (1.16–2.43)	0.006	1.48 (1.03–2.13)	0.034
Water Scarcity	Yes vs No	2.63 (1.74–3.98)	<0.001	1.98 (1.36–2.88)	<0.001
Food Insecurity	Yes vs No	2.57 (1.69–3.90)	<0.001	1.84 (1.26–2.68)	0.002
Displacement	Yes vs No	1.41 (0.93–2.15)	0.105	1.29 (0.86–1.94)	0.215
Brushing Frequency	<2/day vs ≥ 2/day	2.95 (1.88–4.63)	<0.001	2.02 (1.38–2.95)	<0.001
Fluoride Toothpaste	No vs Yes	1.52 (1.02–2.27)	0.041	1.39 (0.96–2.01)	0.083

#### High caries (DMFT ≥3).

Water scarcity was significantly associated with high caries (OR 2.63; 95% CI 1.74–3.98; p < 0.001). Food insecurity also demonstrated a significant association (OR 2.57; 95% CI 1.69–3.90; p < 0.001). Participants brushing less than twice daily had increased odds of high caries (OR 2.95; 95% CI 1.88–4.63; p < 0.001). Lower educational attainment (OR 2.15; 95% CI 1.43–3.22; p < 0.001) and lower income (OR 1.68; 95% CI 1.16–2.43; p = 0.006) were also associated with high caries. Rural residence was associated with higher odds (OR 1.90; 95% CI 1.27–2.85; p = 0.002), while district (Charsadda vs Peshawar) and displacement were not statistically significant.

#### Periodontal disease (CPI ≥ 3).

For moderate-to-severe periodontal disease, rural residence (OR 1.54; 95% CI 1.10–2.17; p = 0.012), older age (≥45 years) (OR 1.92; 95% CI 1.33–2.77; p < 0.001), water scarcity (OR 1.98; 95% CI 1.36–2.88; p < 0.001), and food insecurity (OR 1.84; 95% CI 1.26–2.68; p = 0.002) were significantly associated. Lower education, lower income, and sub optimal brushing practices were also associated with increased odds. District differences were not statistically significant.

### Multivariable logistic regression

Multivariable logistic regression models adjusted for district, residence, age, sex, education, income, climate-related exposures, displacement, and oral hygiene practices as shown in Table 2.

**Table 2:** Multivariable logistic regression (Adjusted OR).

Variable	Category	High Caries AOR (95% CI)	p-value	CPI3 AOR (95% CI)	p-value
District	Charsadda vs Peshawar	1.18 (0.79–1.76)	0.419	1.22 (0.84–1.79)	0.290
Residence	Rural vs Urban	1.32 (0.94–1.86)	0.135	1.41 (1.02–1.96)	0.038
Age Group	≥45 vs 18–29	1.72 (1.03–2.87)	0.039	1.88 (1.29–2.73)	0.001
Sex	Female vs Male	1.05 (0.71–1.56)	0.805	1.09 (0.76–1.58)	0.640
Education	<Secondary vs ≥Secondary	1.68 (1.09–2.60)	0.019	1.36 (0.92–2.01)	0.122
Income	<25,000 PKR vs ≥ 25,000	1.51 (1.01–2.28)	0.045	1.44 (0.98–2.11)	0.064
Water Scarcity	Yes vs No	1.89 (1.23–2.90)	0.004	1.67 (1.12–2.48)	0.012
Food Insecurity	Yes vs No	1.74 (1.12–2.69)	0.014	1.59 (1.05–2.41)	0.027
Displacement	Yes vs No	1.29 (0.79–2.10)	0.308	1.18 (0.73–1.90)	0.494
Brushing Frequency	<2/day vs ≥ 2/day	2.12 (1.32–3.40)	0.002	1.74 (1.15–2.63)	0.009
Fluoride Toothpaste	No vs Yes	1.46 (0.93–2.29)	0.100	1.28 (0.83–1.97)	0.210

Models adjusted for all variables listed above. Model fit was assessed using the Hosmer-Lemeshow goodness of fit test (p > 0.05). Multicollinearity was checked using the Variance Inflation Factor (VIF < 2).

Reference categories: Peshawar (District), Urban (Residence), 18–29 years (Age), Male (Sex), Secondary or above (Education), ≥ 25,000 PKR (Income), No exposure categories for climate variables, ≥ 2/day brushing, and Fluoride toothpaste use.

#### High caries (DMFT ≥3).

After adjustment, water scarcity remained independently associated with high caries (AOR 1.89; 95% CI 1.23–2.90; p = 0.004). Food insecurity also remained significant (AOR 1.74; 95% CI 1.12–2.69; p = 0.014). Brushing less than twice daily was independently associated with high caries (AOR 2.12; 95% CI 1.32–3.40; p = 0.002). Lower education (AOR 1.68; 95% CI 1.09–2.60; p = 0.019), lower income (AOR 1.51; 95% CI 1.01–2.28; p = 0.045), and age ≥ 45 years (AOR 1.72; 95% CI 1.03–2.87; p = 0.039) remained associated. Rural residence, district, displacement, and fluoride toothpaste use were not statistically significant in the adjusted model.

#### Periodontal disease (CPI ≥ 3).

For periodontal disease, older age (AOR 1.88; 95% CI 1.29–2.73; p = 0.001), rural residence (AOR 1.41; 95% CI 1.02–1.96; p = 0.038), water scarcity (AOR 1.67; 95% CI 1.12–2.48; p = 0.012), food insecurity (AOR 1.59; 95% CI 1.05–2.41; p = 0.027), and brushing less than twice daily (AOR 1.74; 95% CI 1.15–2.63; p = 0.009) remained independently associated. Education, income, displacement, fluoride toothpaste use, and district were not statistically significant in the adjusted model.

### Principal component analysis

PCA identified two principal components explaining 65% of the total variance. Component 1 (48%) loaded heavily on water scarcity, displacement, and heat stress, representing climate exposure. Component 2 (31%) loaded on poor oral hygiene practices and food insecurity, representing behavioral and nutritional vulnerability, Table 3.

**Table 3:** Principal component analysis loadings.

Variable	Component 1	Component 2
Water scarcity	0.78	0.22
Displacement	0.74	0.19
Heat stress	0.69	0.18
Poor oral hygiene	0.21	0.81
Food insecurity	0.26	0.77

### Overall interpretation

The results demonstrate that climate-related stressors are significantly associated with adverse oral health outcomes, even after controlling for key socioeconomic and behavioral confounders. The clustering of environmental and behavioral risks highlights the multifactorial nature of oral health vulnerability in climate-affected populations.

**Fig 1:**
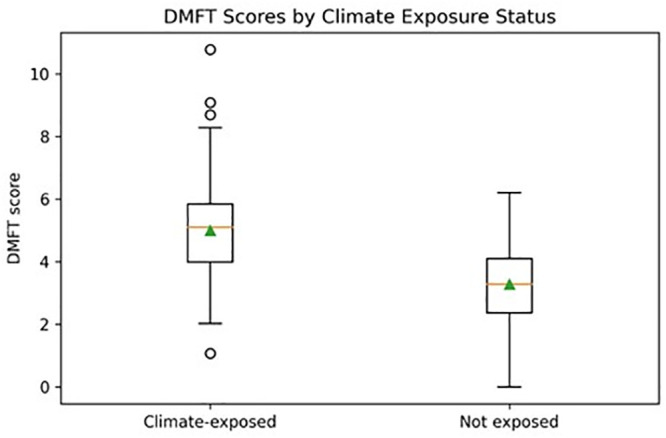
DMFT scores by climate exposure.

**Fig 2:**
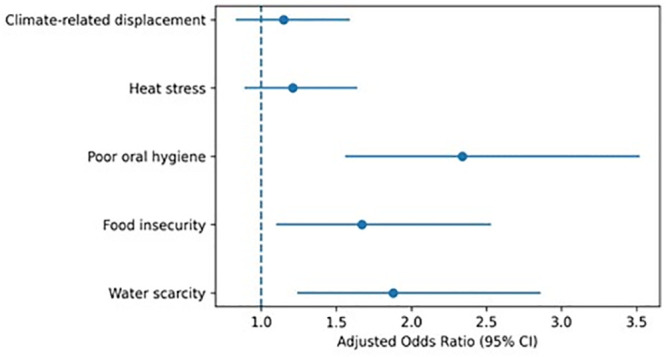
Forest plot of adjusted odds ratios for poor oral health outcomes.

The box plot in Fig 1 illustrates greater vulnerability and higher median DMFT scores among participants exposed to climate-related stressors compared to unexposed participants. The forest plot in Fig 2 summarizes the results of multivariable binary logistic regression models examining predictors of poor oral health outcomes. Water scarcity and food insecurity show adjusted odds ratios greater than 1 with confidence intervals that do not cross the null value, indicating statistically significant associations after adjustment for age, sex, education, income, residence and oral hygiene practices. In contrast, heat stress and climate-related displacement show confidence intervals crossing unity, indicating non-significant associations in adjusted models.

## Discussion

This cross-sectional study examined the association between climate-related exposures and oral health outcomes among adults residing in two districts of Khyber Pakhtunkhwa, Pakistan. The findings demonstrate a high burden of oral disease in the study population, with more than half of participants exhibiting high caries experience (DMFT ≥3) and nearly half presenting with periodontal disease (CPI ≥ 3). Importantly, these adverse oral health outcomes were more prevalent among individuals exposed to climate-related stressors, particularly water scarcity and food insecurity.

After adjustment for socio-demographic and behavioral confounders, water scarcity and food insecurity remained independently associated with poor oral health outcomes, while other climate-related stressors, such as heat exposure and displacement, showed weaker or non-significant associations. Principal component analysis further revealed clustering of environmental stressors with hygiene- and nutrition-related risks, suggesting that climate-related vulnerability operates through interconnected pathways rather than isolated exposures. Collectively, these findings support the interpretation that climate-related exposures are associated with oral health outcomes indirectly, through disruption of established social and behavioral determinants, rather than as direct causal agents.

The observed prevalence of dental caries and periodontal disease in this study is consistent with existing evidence from Pakistan and other LMICs, which documents a substantial unmet burden of oral disease among socioeconomically disadvantaged populations [7,8,25]. The mean DMFT score observed in this study exceeds values reported in some urban Pakistani samples, highlighting the compounded vulnerability of populations residing in peri-urban and rural settings with limited access to preventive and restorative dental care [28,21].

Oral diseases are increasingly recognized as socially patterned conditions, shaped by income, education, access to services, and living environments [22]. In the present study, low household income, limited education, and poor oral hygiene practices were strongly associated with adverse oral health outcomes. These findings reinforce the need to situate oral health within broader frameworks of social determinants and structural inequality, particularly in resource-limited settings.

Water scarcity emerged as one of the strongest predictors of poor oral health outcomes in both bivariate and multivariable analyses. Participants reporting difficulty accessing sufficient water for daily household use exhibited significantly higher odds of high caries burden and periodontal disease. This association persisted after adjustment for income, education, residence, and oral hygiene behaviors. Water availability is a fundamental determinant of oral hygiene practices, including tooth brushing and rinsing, as well as broader sanitation and health behaviors [9]. In contexts of water insecurity, households may prioritize water for drinking and cooking, leading to reduced frequency of oral hygiene practices. Previous studies have linked water scarcity to compromised hygiene behaviors and increased risk of infectious and chronic conditions [11,29]. Although fluoridation status was not assessed in this study due to feasibility constraints, the observed association underscores the importance of reliable water access for maintaining oral health, independent of fluoride exposure.

Food insecurity was independently associated with both high DMFT and periodontal disease in adjusted models. This finding aligns with growing evidence linking food insecurity to poor oral health outcomes through dietary, behavioral, and psycho-social pathways [10,30,31]. Households experiencing food insecurity often rely on inexpensive, energy-dense, and sugar-rich foods, which increase the risk of dental caries [32]. Additionally, nutritional deficiencies associated with food insecurity may impair periodontal health and immune response [33].

Importantly, the persistence of this association after adjustment for income suggests that food insecurity captures dimensions of vulnerability not fully explained by household income alone, such as instability, coping strategies, and psycho-social stress. This distinction is particularly relevant in climate-vulnerable settings, where food insecurity may be driven not only by poverty but also by environmental shocks, seasonal variability, and disrupted supply chains [34].

In contrast to water scarcity and food insecurity, heat stress and climate-related displacement demonstrated weaker and non-significant associations with oral health outcomes after adjustment. These findings suggest that not all climate-related stressors exert equivalent influence on oral health, at least within the time frame and context examined in this study.

Heat exposure may affect health behaviors indirectly through fatigue, dehydration, and reduced physical activity; however, its impact on oral health may be less immediate or more difficult to detect in cross-sectional designs [35]. Similarly, displacement due to environmental events may have heterogeneous effects depending on duration, severity, and access to social support and services. The relatively low prevalence of displacement in this sample may have limited statistical power to detect associations.

The multivariable regression models in this study were explicitly designed to address this concern by adjusting for income, education, residence, age, sex, and oral hygiene practices. The persistence of associations for water scarcity and food insecurity after adjustment suggests that these exposures capture aspects of vulnerability that extend beyond conventional socioeconomic indicators. Nevertheless, the attenuation of some associations after adjustment highlights the importance of cautious interpretation. Climate-related exposures should not be viewed as independent or primary causes of oral disease, but rather as contextual stressors that interact with and amplify existing social and behavioral risk factors. This interpretation aligns with contemporary public health frameworks that conceptualize climate change as a risk multiplier rather than a singular causal agent [36].

The principal component analysis provided additional insight into the structure of vulnerability within the study population. Component 1, characterized by high loading for water scarcity, displacement, and heat exposure, represented a cluster of environmental stressors. Component 2, dominated by poor oral hygiene practices and food insecurity, reflected behavioral and nutritional vulnerability.

The clustering of environmental and behavioral factors underscores the interconnected nature of climate-related risks and oral health determinants. Individuals exposed to environmental stressors were also more likely to experience disruptions in hygiene and nutrition, suggesting cumulative disadvantage rather than isolated risk. Similar clustering patterns have been reported in studies examining climate vulnerability and general health outcomes, reinforcing the relevance of multivariate approaches in understanding complex exposures [37,14]. Importantly, PCA was used in this study as an exploratory tool rather than a basis for causal inference. The findings should therefore be interpreted as descriptive patterns that complement regression analyses and highlight potential pathways for intervention.

The findings of this study are broadly consistent with emerging literature on climate change and oral health, which emphasizes indirect pathways linking environmental stressors to oral disease through social and behavioral mechanisms [16,17]. A recent scoping review highlighted the paucity of empirical studies in this area, particularly in LMIC contexts, and called for population-based research to quantify associations using standard oral health indices [38].

Studies examining food insecurity and oral health have reported associations with caries experience, tooth loss, and unmet dental needs, particularly among low-income populations [10,39]. Similarly, research on water insecurity has demonstrated links to compromised hygiene practices and health outcomes, although oral health has rarely been examined explicitly [11]. The present study contributes to this literature by providing empirical evidence from a climate-vulnerable region in Pakistan, using standardized clinical measures and adjusted analyses.

The findings of this study have important implications for public health policy and climate adaptation strategies in LMICs. Oral health is often excluded from climate-health discourse and adaptation planning, despite its significant contribution to morbidity and quality of life. The observed associations between climate-related exposures and oral health outcomes highlight the need to integrate oral health considerations into broader climate resilience frameworks.

Interventions aimed at improving water security, food systems, and social protection may yield co-benefits for oral health, alongside other health outcomes. For example, ensuring reliable access to safe water can support hygiene practices, while food assistance programs may mitigate dietary risks associated with caries and periodontal disease. Importantly, these interventions address structural determinants rather than individual behaviors alone.

### Strengths and limitations

This study has several strengths, including the use of standardized clinical oral health assessments, adjustment for key confounders, and the application of multivariate techniques to explore clustering of risk factors. The focus on climate-related exposures rather than abstract climate change metrics enhances relevance and interpretability in a cross-sectional design.

However, several limitations must be acknowledged. The cross-sectional nature of the study precludes causal inference and limits the ability to assess temporal relationships. Climate-related exposures were self-reported and may be subject to recall bias. Objective environmental measurements were not available. Additionally, the findings are specific to two districts (Peshawar and Charsadda) in Khyber Pakhtunkhwa, Pakistan and may not be generalizable to other regions.

Future studies should consider longitudinal designs to examine how cumulative exposure to climate-related stressors influences oral health over time. Incorporating objective environmental data, such as water quality and climate indices, may strengthen exposure assessment. Further research is also needed to explore the effectiveness of integrated interventions that address climate vulnerability and oral health simultaneously.

### Conclusion

In conclusion, this study provides empirical evidence that climate-related exposures, particularly water scarcity and food insecurity, are associated with adverse oral health outcomes in a resource-limited Pakistani setting. These associations appear to operate through indirect pathways involving hygiene practices, nutrition, and socioeconomic vulnerability. Integrating oral health into climate adaptation, environmental sustainability, and public health strategies is essential for addressing health inequities in climate-vulnerable populations.

## Supporting information

S1 Data Data. (XLSX)

## Abbreviations

LMICs: Lower Middle Income Countries
DMFT: Decayed, Missing, Filled Teeth
IPCC: Intergovernmental Panel on Climate Change
UNICEF: United Nations International Children’s Emergency Fund
FAO: Food and Agriculture Organization of the United Nations
WHO: World Health Organization
HIV: Human Immunodeficiency Virus
AIDS: Acquired Immunodeficiency Syndrome
AOR: Adjusted Odds Ratio
VIF: Variance Inflation Factor
MCAR: Missing Completely At Random
CPI: Community Periodontal Index
PCA: Principal Component Analysis.
